# Computerized Working Memory Training in Remission From Major Depressive Disorder: Effects on Emotional Working Memory, Processing Speed, Executive Functions, and Associations With Symptoms

**DOI:** 10.3389/fnbeh.2022.887596

**Published:** 2022-06-27

**Authors:** Eivind Haga Ronold, Jutta Joormann, Åsa Hammar

**Affiliations:** ^1^Department of Biological and Medical Psychology, University of Bergen, Bergen, Norway; ^2^Division of Psychiatry, Haukeland University Hospital, University of Bergen, Bergen, Norway; ^3^Department of Psychology, Yale University, New Haven, CT, United States

**Keywords:** computerized working memory training, remission, executive functioning, processing speed, emotional WM, rumination

## Abstract

**Introduction:**

Remission from major depressive disorder (MDD) is associated with residual symptoms related to reduced functioning, quality of life, and relapse risk. Previous studies have raised questions about mechanisms involved-in and affected by cognitive training. This study investigated the associations and changes among depressive symptoms, rumination, processing speed (PS), executive functioning (EF), and emotional working memory (e-WM) pre- post computerized working memory training (CWMT).

**Method:**

Twenty-nine remitted participants were included in a pre- post pilot study of within-subject effects of online CWMT. A total of 20 participants completed the intervention and pre- post tests of EF and PS, e-WM, in addition to symptom and rumination measures. Associations between changes in symptoms and cognition were investigated pre- post. Associations between improvements in CWMT, depression history, and changes in cognition were explored. Hypotheses and statistics were preregistered before data were analyzed.

**Results:**

Manipulation of negatively valanced stimuli in e-WM showed an inverse association with rumination pre-intervention, but the association disappeared post-intervention. Cognitive functioning improved in most conditions with largest effects in EF. Symptoms did not change in the remitted sample. CWMT improvements were related to improvements in some aspects of EF and PS, but also to worse self-reported attention. Depression history was related to less improvement in EF.

**Limitations:**

Sample size was small and there was dropout from the study. There was no control group, thus precluding practice and placebo effects and causal relationships.

**Conclusions:**

Computerized WM training improves cognitive functions and could influence associations between e-WM and rumination. This could counteract functional impairment following MDD.

## Introduction

Major depression (MDD) is associated with lower performance in executive functions (EFs) and processing speed (PS). EFs are the collection of interrelated, but distinct cognitive abilities including *switching* between tasks, *updating*, and *inhibition* of proponent responses and distractions (Friedman and Miyake, 2017), integrated with working memory (WM) and abilities such as planning (Diamond, 2013). Lower performance in tests of EF and PS associated with MDD is referred to as cognitive deficits, which are moderate to large in size (Lee et al., 2012; Snyder, 2013; Parkinson et al., 2020), persist in remission (Hasselbalch et al., 2011; Rock et al., 2014; Semkovska et al., 2019), last for years (Årdal and Hammar, 2011; Ronold et al., 2020b), impair everyday functioning (McIntyre et al., 2013; Evans et al., 2014; Nafilyan et al., 2021; Sumiyoshi et al., 2021), and contribute to relapse and new episodes of MDD (Majer et al., 2004; Schmid and Hammar, 2013; Zajecka, 2013). Evidence-based treatments such as psycho- and pharmacotherapy do not normalize cognitive deficits in remission (Keefe et al., 2014; Rosenblat et al., 2015; Bernhardt et al., 2019; Groves et al., 2021), and deficits can reduce the effects of treatments (McLennan and Mathias, 2010; Groves et al., 2018). Although treatments such as psychopharmacology improve cognition in acute states of MDD (Gorlyn et al., 2015), there is need for interventions for the remitted phase of MDD. Promising new interventions are being investigated with regard to cognitive function (Miskowiak et al., 2022), but knowledge on how and for whom these interventions work is important. In sum, interventions improving cognition in remission from MDD are urgently needed.

The trajectory of cognitive deficits is still being investigated. Deficits are larger during an active episode of MDD, compared to when in remission (Douglas and Porter, 2009; Snyder, 2013; Bernhardt et al., 2019; Semkovska et al., 2019), suggesting *state effects* from depressive symptoms on cognitive functioning. Another perspective is that deficits could exist as a vulnerability before the onset, of MDD, as *traits*. Also, deficits could be a result of depression history or significant stress, through neurotoxic effects and thus exacerbate, as suggested by the *scar hypothesis* (for reviews refer to Hasselbalch et al., 2011; Allott et al., 2016; Ahern et al., 2019). A recent review by Hammar et al. (2022) found support for all these perspectives, relative to the cognitive functions measured. PS could be more state- or scar-related, whereas some EFs could be traits. However, the literature is inconclusive. Cognitive deficits are a major health challenge (Nafilyan et al., 2021), partly on account of the high (Mueller et al., 2013), cumulative relapse rate in MDD (Kessing and Andersen, 2017), and the link between MDD and dementia (Woolf et al., 2021; Hammar et al., 2022). Thus, it is paramount to investigate interventions to improve cognitive deficits for future healthcare needs.

The interrelations between mood symptoms and cognitive functions could vary relative to the domain assessed, as shown above. PS entails how rapidly various tasks can be performed, is associated with current or previous MDD, and is foundational for higher functions such as EF (Salthouse, 1996; Porter et al., 2015). EF is a collection of abilities that show more persistent deficits following symptom remission (Snyder, 2013). According to Nigg et al. (2017), PS and EF relate differently to psychopathology. Supporting this, PS is suggested to be more state-related, whereas EF is more trait-related, in *young* patients with MDD (Douglas and Porter, 2009; Lee et al., 2012). The EF *Inhibition* is sometimes seen as relatively independent of symptoms, whereas *switching* seems to show more associations to MDD (Schmid and Hammar, 2013; Ronold et al., 2020b; Liu et al., 2021). Thus, deficits in EF and PS could have different etiological implications. Tests, however, capture common and distinct EF to a varying degree, a concept commonly conceptualized as task impurity (Snyder et al., 2015), precluding associations between EF and symptoms in the literature. Recently, different conditions of the Stroop and Trail Making Test (TMT) were used in a systematic review investigating PS and EF in MDD, with evidence for deficits in *both functions* (Nuño et al., 2021). Stroop and TMT differentiate between EF and PS and could inform on associations between symptoms and cognitive functions before and following cognitive training (Motter et al., 2019), establishing potential targets for cognitive remediation interventions.

Since recurrence and relapse are more common than single episodes of MDD (Solomon et al., 2000), identifying causes and cures for relapse are vital. Rumination is an emotion regulation strategy which involves self-related, repeated negative thinking about past events (Watkins and Roberts, 2020; Taylor and Snyder, 2021). Rumination related to MDD is measured in several ways, with many studies using the Ruminative Responses Scale (Watkins and Roberts, 2020), measuring general tendency for rumination *in response to depressive mood*. This *depressive rumination* shows large associations with other depressive symptoms (Zetsche et al., 2018) and could contribute to depressive state effects on cognition. Neurotic rumination, measured by the Rumination Reflection Questionnaire (Trapnell and Campbell, 1999), is less studied, measures general tendency for *rumination independent of depressive mood*, and has shown different associations with cognitive functions than the Ruminative Responses Scale (Gustavson et al., 2020; Ronold et al., 2020b). Rumination is consistently associated with relapse, increased symptoms, recurrence, and new episodes of MDD (Nolen-Hoeksema et al., 2008; Aker et al., 2014; Timm et al., 2017; Figueroa et al., 2019; Ronold et al., 2020a; Jandric et al., 2021; Taylor and Snyder, 2021). Deficits in EF, particularly inhibition of negative material, are suggested to contribute to this (Joormann and Gotlib, 2010; Ahern et al., 2019). However, meta-studies find small associations between rumination and shifting, inhibition, and WM discarding (Vălenaş and Szentágotai-Tătar, 2017; Yang et al., 2017; Zetsche et al., 2018). Associations between different forms of rumination, depressive symptoms, EF, and PS have not been studied extensively (Schwert et al., 2017; Gustavson et al., 2020) and could inform on the profile of residual cognitive functions following MDD, and how these can be remediated.

Different cognitive functions could influence the tendency to ruminate. Cognitive processing of emotional material (hot cognition) could show stronger associations with rumination (Roiser and Sahakian, 2013; Joormann and Stanton, 2016; Ahern et al., 2019). Meta-studies find limited effects of emotional valence on associations between EF tests and rumination (Vălenaş and Szentágotai-Tătar, 2017; Yang et al., 2017), but there is evidence for persisting emotional bias in remission (Semkovska et al., 2019). One meta-study concluded that discarding of negative information from emotional working memory (e-WM) could be related to rumination (Zetsche et al., 2018). Diverse methodology could preclude associations between (hot) EF and rumination in the literature. Deficits in manipulation of negative stimuli in e-WM have shown associations with rumination (Joormann et al., 2011; Ronold et al., 2020a). In addition, one study found that rumination was associated with both PS *and* EF (Schwert et al., 2017). Thus, it is important to measure associations among symptoms, rumination, and e-WM, PS, EF, how interventions influence associations, and thus potentially improve functions.

Subjectively reported cognitive functions could be important residual symptoms in remission from MDD. Self-reported, subjective deficits persist in remission following first episode MDD (Schmid and Hammar, 2021) and appear to be largely independent of objective cognitive tests (Snyder et al., 2020). Subjective cognition has been found to be a potential predictor of quality of life, functioning, and relapse, following MDD (Sumiyoshi et al., 2021). Thus, intervention targeting subjective cognition is important. One recent study by Lengvenyte et al. (2020) found the improvements in depressive symptoms, particularly self-rated attention, in a remitted bipolar group following computerized WM training (CWMT). Thus, it is important to further assess whether new interventions such as CWMT could improve self-rated aspects of cognition and residual MDD symptoms.

Given the persisting residual symptoms described above, research on new interventions targeting residual cognitive symptoms during MDD remission is needed. Computerized cognitive training paradigms, such as CWMT, have been suggested as an intervention in remission from MDD (Motter et al., 2016; Koster et al., 2017). Such interventions have the advantages of being relatively cost efficient, engaging, and can be performed from home. WM is a function closely related to the EF updating and entails manipulation of material in short-term memory (Miyake et al., 2000). WM has been suggested as a target for enhancing cognition through CWMT (Webb et al., 2018; Motter et al., 2019). Recent studies indicate that the improvements appear in depressive symptoms, EF, and PS, following training (Barkus, 2020; Launder et al., 2021; Woolf et al., 2021), but results are mixed with studies including higher symptom levels showing most symptom improvement (Legemaat et al., 2021), and the underlying mechanisms in improvements are largely unknown (Webb et al., 2018; Motter et al., 2019; Ferrari et al., 2021). Given these mixed results (Lengvenyte et al., 2020; Legemaat et al., 2021), investigating depressive symptoms following CWMT with particular focus on subjective cognition seem important. Since emotional processing, subjective difficulties, cognitive deficits, and rumination all contribute to relapse and recurrence of MDD, studies exploring how CWMT influences these processes are warranted.

The aim of this study was to investigate how residual cognitive symptoms in remission from MDD are effected by CWMT. Previous research on most of the current sample concluded that CWMT was well accepted and improved EF and PS (Hammar et al., 2020). Given the clinical significance of residual cognitive symptoms outlined above, it is crucial to study how interventions affect such symptoms in remission from MDD. Improvements and altered associations among cognitive functions, symptoms, following CWMT could support the clinical significance of such interventions and suggest targets with future treatments.

*Pre-Intervention:* it was expected that emotional working memory for negative stimuli would show negative associations with rumination (Joormann et al., 2011; Ronold et al., 2020a). Processing speed and switching were expected to be associated with depressive symptoms (state/scar) whereas inhibition was expected to be independent (trait) of depressive symptoms (Ronold et al., 2020b).

*Post-Intervention:* cognitive functioning and symptoms of depression and rumination were expected to improve, while the associations between cognitive functioning and symptoms of depression and rumination, were expected to decrease.

In addition, associations between the CWMT intervention improvement, cognitive functions, and clinical variables were explored.

## Materials and Methods

The current data were collected as a part of a pilot study on the feasibility of CWMT in remission from depression (Hammar et al., 2020) and are a pre–post-study of how CWMT influences e-WM, EF, PS, and associations with depressive symptoms. Hypotheses and methods were preregistered on Open Science Framework (https://osf.io/vpxgw) before analysis, but after data collection was completed.

### Participants and Procedure

Participants were recruited through an outpatient clinic for affective disorders at the Psychiatric unit of Haukeland University Hospital and through advertisements and previous research projects. A total of 29 participants were included in the study, and of these, 20 completed the intervention and retest. Inclusion criteria were age (20–60), previous treatment of MDD, currently remission, or recovery as indicated by ≤ 12 on The Montgomery Åsberg Depression Rating Scale (MADRS; Montgomery and Åsberg, 1979). Exclusion criteria were brain damage/pathology, severe somatic disorders, alcohol, or substance abuse. The Mini-International Psychiatric Structural Interview (Sheehan et al., 1998) was used to confirm diagnosis and history of MDD, and screen for exclusion criteria. Other clinical data were assessed by a structured form developed for the study. A psychologist or a psychiatric nurse assessed inclusion criteria at the outpatient clinic for affective disorders or the neuropsychological clinic at the University of Bergen. Neuropsychological and experimental assessment was performed at the neuropsychological clinic, both preceding (T1) and following the intervention (T2). The CWMT intervention was delivered at home online on a computer or tablet. Participants started training within 2 weeks following assessment. After the intervention participants were invited to a posttest assessment of e-WM, EF, and PS, depressive symptoms, and rumination.

#### Missing Data and Participant Flow

**Figure 1:**
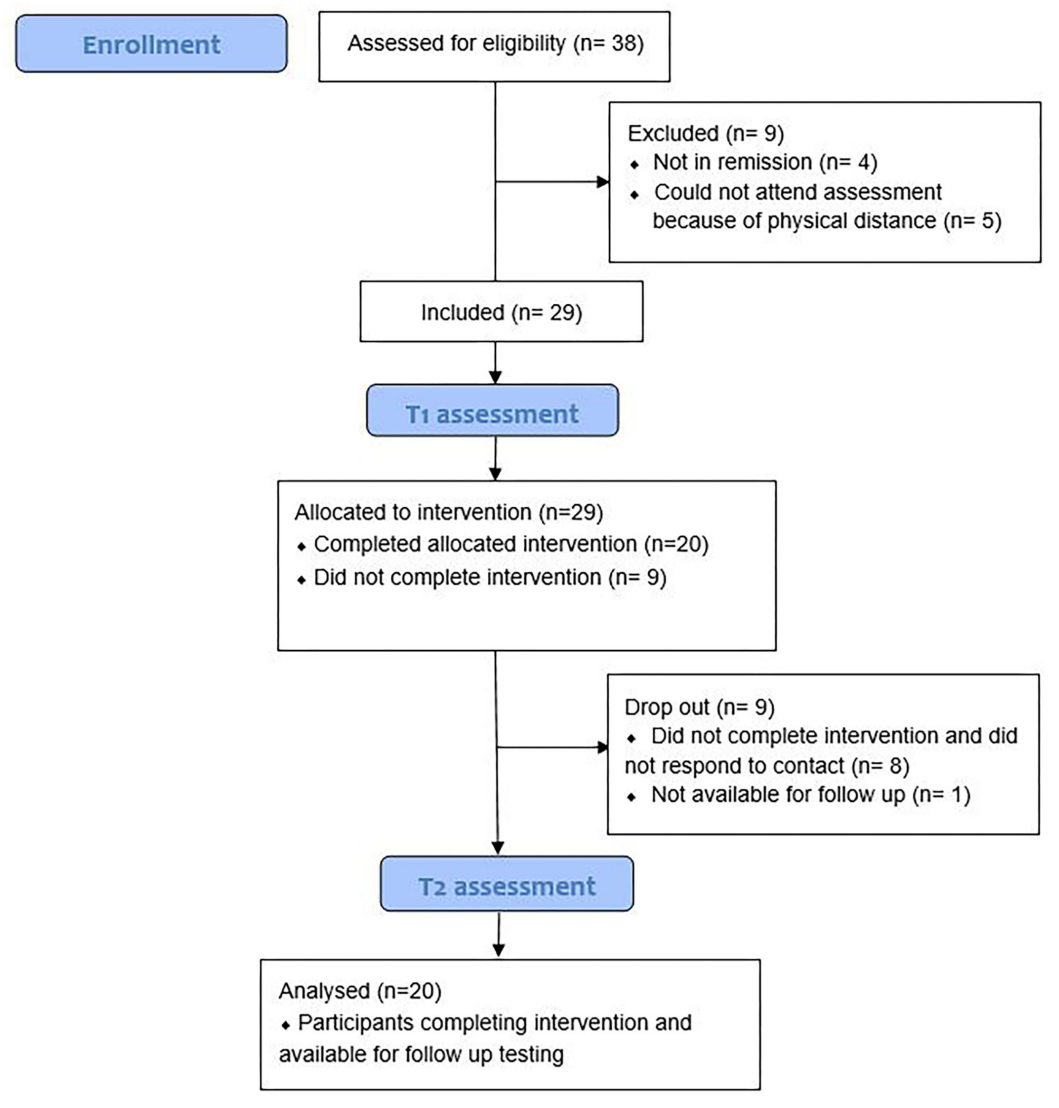
Flow chart of participants.

Due to technical difficulties, T1 data for e-WM were missing for two participants at T1, and three participants at T2, thus resulting in pre–post-data for 16 participants. One participant was missing MADRS at T1, and four missed the item measuring self rated attention. A total of two participants were missing MADRS at T2, thus resulting in pre–post-data for 18. For demographical characteristics, refer to Table 1. For more information about participant flow, refer to Figure 1.

**Table 1:** Demographic characteristics and symptoms.

	**T1 *n* = (m/f)**	**T2 *n* = (m/f)**			
Mean (SD)	*n* = 29 (10/19)	*n* = 20 (8/12)			
Age	36.207 (10.896)	37.3 (12.27)			
Education	16.53 (1.792)	16.55 (1.731)			
IQ	115.828 (8.06)	*	*t*/*Z*	*p* (one-tailed)	E. s.
MADRS	6 (4.35) *n* = 28	8.78 (8.742) *n* = 18	*Z* = 0.971	*p* = 0.166	*r* = 0.161
MADRS SCI	1.292 (1.97) *n* = 24	1.54 (1.33) *n* = 18	*Z* = 0.79	*p* = 0.215	*r* = 0.13
RRS	48.07 (13.48)	46.75 (14.664)	*t* = 0.38	*p* = 0.355	*d* = 0.085
RRQ-r	40.928 (9.561) *n* = 28	40.8 (9.638)	*t* = 0.763	*p* = 0.226	*d* = 0.171

*m/f, Male/Female; MADRS, Montgomery Åsberg Depression Rating Scale; SCI, Subjective cognition item; RRS, Ruminative Responses Scale; RRQ-r, Rumination Reflection Questionnaire rumination*.

### Computerized Working Memory Training Intervention

Cogmed™ (Pearson, 2022) was chosen as the CWMT intervention. It is a commercial CWMT program that has several advantages: It is performed online, so participants can use it at home, it has a considerable duration, with incremental difficulty depending on past performance (successfully completed tasks), both aspects that are considered important for successful CWMT (Koster et al., 2017; Launder et al., 2021). Cogmed targets “cold cognition,” i.e., working memory span. The program consisted of several different number, letter, and spatial, forward and backward sequencing tasks. For example, Letters are read out loud, while one of several rotating bubbles lights up on screen, participants remember the sequence of the lighted bubbles, clicking on the corresponding bubbles sequentially following presentation. In another task, participants attend to a sequence of numbers read out and then click on the numbers in reverse sequence on screen. In a different task, participants remember the sequence of panels on a cube that light up one by one, then clicking on the panels in the correct sequence after presentation. The intervention consisted of 25 sessions, lasting ~5 weeks, with five weekly 30–40-min sessions. In addition, there was weekly telephone contact between participants and a trained Cogmed coach, discussing progress, motivation, and questions related to the training.

### Neuropsychological Assessment

All participants completed a neuropsychological test battery pre- and post-intervention measuring general intellectual ability, EF, and PS. General intellectual ability was measured by the Norwegian version of the Wechsler Abbreviated Scale of Intelligence (Wechsler, 1999), to better describe the sample and potentially explain dropout (refer to Table 1). The Delis–Kaplan Executive Function System (Delis et al., 2001) subtests TMT, and the Color Word Interference Test (CWIT), an updated version of the Stroop task, was used to measure PS and EF. The TMT is a paper and pencil task consisting of five conditions. The CWIT consists of four conditions with verbal responding to visual stimuli.

#### Measurements of PS

The first condition of TMT, *visual scanning*, consists of marking out symbols. The following two conditions, *number* and *letter* sequencing, require sequential marking of numbers and letters, respectively. These three conditions measure PS. Finally, the fifth condition, *motor speed*, consists of swiftly marking a line and is a measure of motor speed. The first conditions of the CWIT, *color* and *word reading*, measure PS.

#### Measurements of EF

Color Word Interference Test and TMT measure inhibition and switching. In the fourth condition of the TMT, participants switch between marking letters and numbers in rising alphabetical and sequential order, measuring the EF *switching*. The third condition of CWIT consists of naming the printed color of an incongruently spelled color word, modeled on the classical Stroop effect (Stroop, 1935), and measure the EF inhibition. The last condition of CWIT alternates between condition three and condition two and thus measures both EFs inhibition *and* switching (Inhibition Switching), or arguably more of the common than specific aspects of EF.

### Emotional WM Paradigm

**Figure 2:**
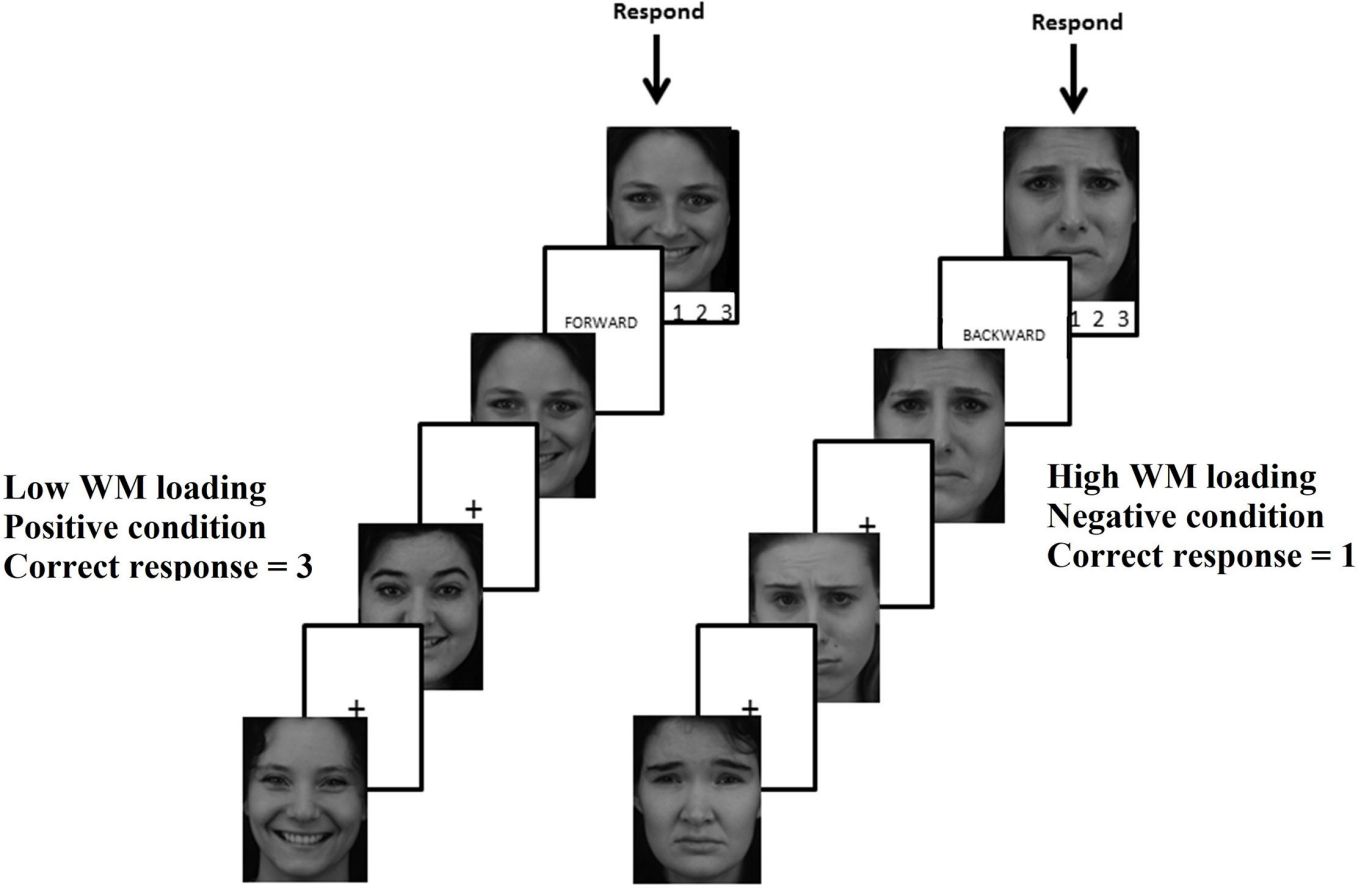
Emotional working memory paradigm using pictures from the Karolinska Directed Emotional Faces.

Emotional working memory was measured by a computerized paradigm pre–post-intervention (refer to Figure 2). Participants were presented with a series of three happy or sad faces (Lundqvist et al., 1998) and then instructed to hold the sequence in mind in either a forward- (low WM loading) or *manipulate* in a backward (high WM loading) sequence. Finally, participants were shown one of the three faces and asked to specify where in the (rotated) picture sequence, the face was presented by pressing key “1,” “2,” or “3.” The experiment was run in E-prime (version 2), and percent accuracy was used as an outcome measure. For further description of the paradigm, refer to Ronold et al. (2020a).

### Self-Report Scales

Several self-report measures were administered pre- and post-intervention. The Ruminative Responses Scale (RRS) and Rumination Reflection Questionnaire (RRQ) measured depressive and neurotic rumination, respectively. Depression was measured post-test by MADRS self-report.

### Ruminative Responses Scale

The Norwegian version of the form RRS (Treynor et al., 2003) was administered pre- and post-intervention. It consisted of 22 questions in a four-point scale from 1 “almost never” to 4 “almost always” (22–88 range) and asked participants lifetime proclivity for rumination when “feeling sad or depressed,” e.g., “think about how sad you feel.”

### Rumination Reflection Questionnaire – Rumination Subscale

The rumination subscale from the Norwegian version of the RRQ was used to measure lifetime proclivity for self- ruminative though, independent of depressive mood, e.g., “My attention is often focused on aspects of myself I wish I'd stop thinking about.” Here, 12 items are rated on a five-point scale ranging from 1 “strongly disagree” to 5 “strongly agree” (12–60 range). The scale has been shown to correlate with the personality trait neuroticism and could thus measure *neurotic rumination* relatively independent of mood (Trapnell and Campbell, 1999).

### The Montgomery Åsberg Depression Rating, and Self-Report Scale

Clinician administered MADRS was used to assess depression pre-intervention. Following the intervention, the Montgomery Åsberg Depression Rating Scale Self-report (Svanborg and Åsberg, 2001) was used to assess depression. This measure is comparable to the MADRS with the exception of one item (item 1) that assess apparent sadness. Depressive symptoms are scored on a seven-point scale from zero to six (range 0–54 relative to MADRS 0–60) and have proven to be useful and sensitive to change in depressive symptoms in clinical trials (Cunningham et al., 2011). Items 6 and 5 measure self-subjectively experienced difficulties with attention on MADRS and MADRS Scale Self-report, respectively.

### Ethics and Compensation

All participants provided informed signed consent prior to participating in the study. The study was conducted in accordance with the Helsinki Declaration of Ethical Research regarding Ethical Principles for Medical Research Involving Human Subjects (World Medical Association, 2013) and was approved by the committee for ethical research in Western Norway (2014/1079), in addition to the Norwegian Data Inspectorate. Participants were awarded with a gift card valued at 400 Norwegian Kroner (~50 dollars), for their participation. In addition, participants were given access to the CWMT program free of charge.

### Data Scoring and Statistical Analysis

E-Data aid (version 2) was used to calculate accuracy in the e-WM paradigm and exported to a statistical program used for all analyses (version 25). For the neuropsychological tests, raw scores consisting of seconds to complete tasks were used. Data were screened for outliers using boxplots and histograms. Normality was assessed by Kolmogorov–Smirnov test, and non-parametric tests were used when assumptions were violated. To investigate the pre-intervention hypotheses, associations between cognition and symptoms post-intervention, in addition to the explorative analyses, the bivariate correlation coefficient, Pearson's *r* was used. To investigate post-intervention hypothesis and attrition, paired sample *t*-tests were used, with Wilcox signed rank test as a non-parametric alternative. Spearman's rank-order correlation ρ was calculated for non-parametric data. To avoid risk for type 2 errors, Bonferroni correction was not strictly implemented (Nakagawa, 2004), but it has been noted which improvements remained significant after this correction (refer to Table 2). Significance level was set at *p* = 0.05 (Bonferroni correction for Table 2 = 0.05/16 = 0.003), and one-tailed significance levels were used to assess pre-registered hypotheses. Effect sizes (Cohen's *d*), where reported, for non-parametric tests *r*, were calculated from the following formula *r* = Z/√ total number of observations. Effect sizes were reported as small (*d* = 0.2, *r*/ρ =0.1), moderate (*d* = 0.5, *r/*ρ = 0.3), and large (*d* = 0.8, *r/* ρ = 0.5) according to Cohen (1988).

**Table 2:** Changes in e-WM, PS and EF from T1 to T2.

	**T1 *n* = 29**	**T2 *n* = 20**	***t*/*Z***	***p* (one-tailed)**	**E. s**.
e-WM*	*M (SD)*	*M (SD)*			
Low WM negative	66.2% (20%)	74.5% (17.5%)	*Z* = 2.143	*p* = 0.016	*r* = 0.378
High WM negative	62% (22.6%)	70.8% (17.9%)	*t* = 2.202	*p* = 0.022	*d* = 0.55
Low WM positive	65.2% (21%)	74.3% (20.9%)	*Z* = 2.55	*p* = 0.006	*r* = 0.45
High WM positive	64.7%(21.9%)	68.3% (13.7%)	*Z* = 2.045	*p* = 0.021	*r* = 0.361
Trail Making Test**					
Visual scanning	20.28 (7.755)	17.4 (5.413)	*Z* = 1.544	*p* = 0.062	*r* = 0.244
Number Sequencing	26.07 (8.606)	23.5 (8.935)	*Z* = 1.941	*p* = 0.026	*r* = 0.306
Letter sequencing	24.72 (9.42)	22.50 (10.08)	*Z* = 2.035	*p* = 0.021	*r* = 0.322
Number Letter Switching	69.03 (20.36)	55.8 (18.62)	*t* = 2.347	*p* = 0.002^b^	*d* = 0.748
Contrast LNS Switching	46.47 (17.24)	35.32 (15.69)	*t* = 2.908	*p* = 0.005	*d* = 0.65
Motor speed	19.17 (9.54)	18.50 (7.81)	*Z* = 1.246	*p* = 0.105	*r* = 0.197
Color Word Interference	**				
Color Naming	28.97 (4.45)	27.75 (5.24)	*t* = 2.666	*p* = 0.008	*d* = 0.596
Word Reading	21.45 (3.501)	20.95 (3.65)	*t* = 2.58	*p* = 0.009	*d* = 0.577
Inhibition	50.66 (10.25	44.05 (8.501)	*t* = 4.25	*p* < 0.001^b^	*d* = 0.95
Contrast Inhibition	25.44 (8.805)	19.70 (6.118)	*t* = 3.16	*p* = 0.003^b^	*d* = 0.706
Inhibition/Switching	53.45 (7.822)	49.1 (8.771)	*t* = 2.102	*p* = 0.025	*d* = 0.47
Contrast I/S	19.76 (7.135)	18.183 (5.557)	*t* = 0.536	*p* = 0.299	*d* = 0.120

* *Means for the whole sample, pre- n = 28 post- n = 17, accuracy in percent.*

** *Means for the whole sample, accuracy in seconds, high score = poor performance, LNS = Number Letter Switching I/S = Inhibition/Switching.*

b *Significant after Bonferroni correction*.

### Z-Transformations, Contrast, Improvement, Composite, and Change Scores

*Z-score transformation* of MADRS pre- and post-intervention was calculated to compare the change in depression from pre- to post-test. *A depression history composite* was computed from the following Z-transformed depression and rumination measures (T1 MADRS + T2 MADRS + MDD length in months + number of MDD episodes + T1 RRS + T2 RRS + T1 RRQ + T2 RRQ)/8. *Change scores* for neuropsychological tests, RRS, RRQ, and MADRS were calculated by subtracting T2 scores from T1 scores. *Change scores* in e-WM were calculated by subtracting scores at T1 from scores at T2. Thus, positive values indicated post-intervention improvements. *Separating EF from PS:* Contrast scores were calculated to separate EF from PS: TMT Switching (TMT Switching contrast = Number-Letter Switching – (Visual Scanning + Letter Sequencing + Number Sequencing + Motor Speed)/4), Inhibition (Inhibition Contrast = Inhibition – (Color Naming + Word Reading)/2) CWIT Switching condition (CWIT Switching Contrast = Inhibition/Switching – (Color Naming + Word Reading + Inhibition)/3). *CWMT improvement score:* Improvement in CWMT was calculated through the formula: highest performance score (i.e., session with most completed tasks) – starting performance score/starting performance score.

## Results

### Attrition Analysis

There were no significant differences in demographic characteristics in Table 1, depressive symptoms, EF, PS, e-WM, with small effect sizes, between participants who completed the intervention and participants that dropped out, suggesting that attrition effects were limited.

### Pre-Intervention Hypotheses

#### Rumination and e-WM

Negative moderate correlations between depressive rumination (RRS total score) and e-WM in the high WM load negative condition did not appear (*r* = −0.076 *n* = 27 *p* = 0.354, one-sided). Exploring this further, the expected moderate negative association appeared between neurotic rumination (RRQ rumination) and e-WM in the high WM loading negative condition *r* = −0.381 *n* = 26 *p* = 0.028 (one-sided). There was no association between MADRS and depressive rumination ρ = 0.052 *n* = 28 *p* =0.396 (one-sided). In sum, there was no association between depressive, but a moderate negative relationship between e-WM and neurotic rumination. Depressive symptoms did not correlate with rumination.

#### Depressive Symptoms and PS

Depressive symptoms (MADRS) showed small negative associations with the Color Naming and Word Reading conditions of CWIT (ρ = −0.290 and −0.234), and Visual Scanning, Letter and Number Sequencing, and Motor Speed conditions of the TMT (ranging from ρ = −0.134 to −0.250). In sum, positive associations between depressive symptoms and PS did not appear.

#### Depressive Symptoms and EF

Color Word Interference Test inhibition was independent of depressive symptoms ρ = −0.038 *n* = 28 *p* = 0.424 (one-tailed). Opposite associations were also found for EF and depressive symptoms with moderate negative association between CWIT Inhibition Switching, a small negative association between TMT switching (ρ = −0.385 and ρ = −0.256, respectively), and smaller associations with contrast scores (ρ = −0.194 and ρ = −0.038, respectively). In sum, positive associations between depressive symptoms and switching did not appear. Inhibition was independent of depressive symptoms.

### Post-Intervention Findings

#### Improvements in e-WM

Participants showed significant moderate e-WM improvements (refer to Table 2). The negative (sad faces) low WM loading condition showed improvements, from pre- (*Md* = 62.25%) to post- (*Md* = 75%), and the high WM loading maintain condition, improvements from pre- (*M* = 60.38%) to post- (*M* = 69.56%) intervention. In the positive conditions (happy faces), improvements appeared in the low WM loading condition, changing from pre- (*Md* = 71%) to post- (*Md* = 79%) intervention and in the high WM loading condition from pre- (*Md* = 58%), to post- (*Md* = 67%) intervention. Thus, participants improved across all e-WM conditions following CWMT.

#### Improvements in PS

Trail Making Test scores showed small to moderate improvements in PS following CWMT (refer to Table 2). TMT Number and Letter Sequencing showed significant moderate improvements from pre- (*Md* = 24) to post- (*Md* = 22), and pre- (*Md* = 23.5) to post- (*Md* = 21) intervention, respectively. TMT Visual Scanning showed small non-significant changes pre- (*Md* = 19) to post- (*Md* = 16.5) intervention, and Motor Speed showed small non-significant changes pre- (*Md* = 16) to post- (*Md* = 16) intervention. Moderate significant improvements in PS appeared for the CWIT scores, with improvements in both Color Naming pre- (*M* = 29.5) to post- (*M* = 27.75) intervention, and Word Reading pre- (*M* = 22) to post- (*M* = 20.95) intervention. Thus, participants showed moderate significant improvements in PS, except in Visual Scanning and Motor Speed.

#### Improvements in EF

Trail Making Test showed moderate to large improvements in switching. The Number Letter Switching condition showed a moderate to large improvement pre- (*M* = 71.75), to post- (*M* = 55.8) intervention, and this effect was also moderate in the contrast score controlling for processing speed, pre- (*M* = 48.613) to post- (*M* = 35.33) intervention. Large improvements in inhibition appeared on the CWIT pre- (*M* = 49.1) to post-(*M* = 44.05)intervention. A significant moderate to large effect remained in a contrast score controlling for processing speed pre- (*M* = 23.325) to post-(*M* = 19.7)intervention. In the CWIT condition, measuring inhibition and switching showed small to moderate significant improvement pre- (*M* =52.7) to post- (*M* = 49.1) intervention. This effect, however, was non-significant, and small, in a contrast score controlling for processing speed. Thus, participants improved across most EF conditions following CWMT, with largest improvements in inhibition.

#### Change in Depressive Symptoms and Rumination

There were no significant improvements in depressive symptoms measured by MADRS, MADRS subjective attention, RRS, and RRQ rumination scores, pre–post-intervention (refer to Table 1). Overall, participants did not show significant improvements in symptoms following CWMT, although initial values on MADRS were low with low variance.

#### Associations Between Symptoms and Cognitive Functioning Post-Intervention

The association between neurotic rumination and the negative high WM loading condition was small and no longer significant *r* = 0.052 *n* = 17 *p* = 0.421 (one-tailed) post-CWMT. TMT Number Letter Switching showed positive associations with depressive symptoms at T2, with moderate non-significant correlations between TMT Number Letter Switching and MADRS at T2 ρ = 0.331 *n* = 18 *p* = 0.090 (one-tailed), and significant correlations with the switching contrast score ρ = 0.416 *n* = 18 *p* = 0.043 (one-tailed). In addition, MADRS and depressive rumination (RRS) showed large correlations post-intervention ρ = 0.732 *n* = 18 *p* < 0.001 (one-tailed). Thus, associations between e-WM and rumination decreased following the intervention, whereas associations between depression symptom load and EF, and depressive rumination, appeared post-intervention.

### Explorative Hypotheses

#### Associations Between CWMT Improvement and Improvements in PS

The CWMT improvement score was associated with improvements in PS change scores; TMT- Visual Scanning ρ = 0.419 *n* = 20 *p* = 0.033 (one-tailed) and Letter Sequencing *r* = 0.378 *n* = 20 *p* = 0.05 (one-tailed). Thus, there were moderate associations between CWMT improvement and some tests of PS.

#### Associations Between CWMT Improvement and Improvements in EF

The CWMT improvement score was associated with the Inhibition Switching change score *r* = 0.469 *n* = 20 *p* = 0.018 (one-tailed), and the change contrast score in this condition ρ = 0.407 *n* = 20 *p* = 0.037 (one-tailed). Thus, there were moderate associations between inhibition switching and improvements in CWMT.

#### Associations Between CWMT Improvement and Improvements in Symptoms

The CWMT improvement score was not related to changes in symptoms, except for a negative association with change in MADRS self-assessed cognition ρ = −0.406 *n* = 18 *p* = 0.048 (one-tailed). Thus, there were a moderate negative associations between improvement in CWMT and self-assessed attention change score, suggesting exacerbation, from pre- to post-intervention.

#### Associations Between Duration and Strength of MDD, Change in EF, Rumination, and e-WM

A depression load composite score consisting of all symptoms, number, and length of depressive episodes was calculated. The composite score was related to changes in EF: A moderate negative correlation to change score Inhibition Switching ρ = −0.404 *n* = 16, *p* = 0.06 (one-tailed), with greater significant associations with the change in contrast score in this condition ρ = −0.499 *n* = 16, *p* = 0.024 (one-tailed). Thus, participants with longer and more severe episodes of MDD showed less improvement in EF.

## Discussion

The aim of this study was to investigate how a computerized working memory training intervention affected residual cognitive symptoms in remission from MDD. This study investigated association among depressive symptoms and rumination, processing speed, executive functions, and emotional WM, and how these are affected by computerized working memory training. The results show both broad and specific effects of computerized working memory training. Broadly, main findings support the successful implementation of computerized working memory training for improving processing speed, executive functions, and emotional working memory. Specifically, associations between rumination and negative high loading working memory deficits could be altered by the intervention, and improvements appear larger in executive functions. Depression history could influence the effects of working memory training on executive functions.

### Pre-Intervention Hypotheses

There was partial support for an association between rumination and e-WM. Neurotic rumination showed a moderate negative association with negative high WM loading material in e-WM, supporting associations between rumination and difficulties in manipulation of negative material in WM (Joormann et al., 2011; Ronold et al., 2020a). Inhibition was independent of depressive symptoms as expected (Snyder, 2013; Ronold et al., 2020b). The lack of association between depressive rumination measured by RRS and e-WM could be due to the low level of depressive symptoms in the group at T1 (remission as inclusion criteria) and could help explain the lack of depressive state effects on cognitive functions such as PS and switching. The direction of associations between depressive symptoms and cognition went opposite of predictions, and from what is usually reported in the literature (McDermott and Ebmeier, 2009: Snyder, 2013; Semkovska et al., 2019). It is thus likely that participants must show higher levels of depressive symptoms for state effects to appear (Dotson et al., 2020), which is noteworthy given the current remitted sample with low levels of depressive symptoms. Supporting this were the findings at T2 when symptoms and variance were higher. Here, some of the expected associations between depressive symptoms and switching and rumination were more evident. This could indicate that some associations between symptoms and cognition remain following CWMT and thus are impacted to a limited degree by improvements in cognition, suggestive of trait or scar effects. Thus, e-WM showed expected associations with rumination, whereas EF and PS did not show expected associations with depressive symptoms. Limited support for state effects on cognition in remitted MDD appeared, and inhibition emerged as a possible trait, independent of variations in current depression symptoms, but state effects were probably difficult to detect due to low variance and score of depression in the current sample initially.

### Post-Intervention Hypotheses

Hypotheses about improvements in e-WM, PS, and EF following CWMT were supported. Participants improved across all e-WM conditions, most PS conditions, and most EF conditions, following CWMT. Effects were largest for CWIT Inhibition and TMT Number Letter Switching, and these were the only conditions statistically significant after Bonferroni correction, supporting larger effects for CWMT in EF tasks, and potential for the remediation of trait deficits associated with psychiatric disorders such as MDD. This is in line with stronger associations between EF and WM, relative to PS (McCabe et al., 2010), supporting transfer between CWMT and EF improvements. This could be important since EF deficits are of particular clinical significance (Snyder et al., 2015; Groves et al., 2018). However, the improvements compared to normative data indicated that that only inhibition improved descriptively, i.e., from “low average” to “average” (Delis et al., 2001). Other studies, in contrast, find superior improvements in PS following training (Webb et al., 2018). Of note, improvements were larger than retest effects reported in the literature (Scharfen et al., 2018), which support causal effects of CWMT on both PS and EF. An association between the EF switching, measured by TMT, and depressive symptoms, emerged following CWMT, suggesting that some associations between symptoms and EF does not disappear following CWMT.

Post-intervention, e-WM showed improvements with similar effects across conditions. The correlation between negative high loading WM condition and rumination disappeared following CWMT, as expected. This suggests that improvements in manipulation of negative material in WM, suspected of contributing to rumination (Ronold et al., 2020a), could improve ruminative tendencies. Improvements in hot cognition following CWMT were found, which could improve the clinical course of remission from MDD (Ahern et al., 2019). Processing of emotional faces could be of particular interest due to its implications for social interaction, and improvements could have positive consequences for interpersonal function (Bourke et al., 2010). This study did not find improvements in symptoms, however, precluding the clinical implications of the reduced associations between e-WM and rumination. Thus, the clinical importance of cognitive improvements remains unclear. In addition, improvements in objective cognitive functions did not transfer to subjective ratings of attention, and there were suggestions that CWMT improvements exacerbated self-rated attention. Objective and subjective cognition are largely distinct (Snyder et al., 2020) however, and findings regarding CWMT and symptomatic improvements are mixed (Barkus, 2020; Legemaat et al., 2021). This could suggest that broader interventions (Myklebost et al., 2021), selection, and personalized interventions for individuals and subgroups with varying degrees of residual symptoms are necessary for optimal effects to appear.

Hypothesis about improvements in symptoms was not supported. Thus, transfer effects, beyond cognition, were not found (Grinberg et al., 2021). This is contrary to the findings of Lengvenyte et al. (2020), where the authors found improvements in depressive symptoms including subjective attention, using similar methods and interventions as this study. However, the authors used subjective cognitive complaints as an inclusion criterion. In addition, it is not evident that MADRS is an optimal measure of subjective cognition (Russo et al., 2015). A recent meta-analysis found that improvements in symptoms following cognitive remediation were related to higher baseline symptomatology (Legemaat et al., 2021), which could help explain our findings. This study had low initial depressive symptoms that could have resulted floor effects in improvement, also influencing sensitivity to state effects. However, the main aim of the study was to investigate CWMT effect on residual cognitive symptoms and not mood symptoms *per se*, resulting in the low mood symptom load in the fully remitted sample at inclusion. Sample size also probably affected results. Meta-studies generally find small associations between EF and rumination (Vălenaş and Szentágotai-Tătar, 2017; Yang et al., 2017; Zetsche et al., 2018), and thus, associations might be too small to detect with the current sample. Rumination did not improve, which could suggest that CWMT does not target this function in remission from MDD. Supporting this, a recent randomized controlled trial did not find effects of cognitive control training on rumination (Ferrari et al., 2021). Possibly, effects could only be evident for a subsample of remitted individuals (Hammar et al., 2020; Woolf et al., 2021), with differing degrees of residual symptomatology and cognitive deficits (Paelecke-Habermann et al., 2005; Pu et al., 2018; Ronold et al., 2021). Thus, inclusion through some sort of objective or subjective deficit or criteria could be essential for optimal effects of CWMT to manifest. The CWMT intervention targeted cold cognition. Targeting hot cognition instead might show larger effects on symptoms as suggested by a recent meta-analysis (Sociali et al., 2022). Future research should compare the effects on interventions targeting hot and cold cognition on several clinically relevant residual symptoms to examine utility for relapse prevention.

### Explorative Hypotheses

Explorative analyses found associations between improvements in CWMT and improvements in EF and PS from T1 to T2. Some of the changes in PS and EF showed moderate associations to improvements in CWMT. EF also showed moderate associations to improvements in CWMT, even when controlling for PS. This supports that improvements are related to CWMT and not merely retest effects, and that CWMT improves both EF and PS. In addition, the depression history composite score showed negative associations to the CWIT Inhibition Switching change score, thus supporting an EF deficit associated with depression that could also impair EF improvements from CWMT. This could partly explain the relatively small improvement in this condition and suggests that previous history of illness could impair improvements in parts of the EF domain. These findings could have implications for the timing of interventions and could suggest that some patients may show better training effects for some domains, by utilizing CWMT *early* in their illness (Ahern and Semkovska, 2017). Perhaps, other interventions such as attention bias modification training (Koster and Hoorelbeke, 2015) or interventions targeting hot cognition are more useful than CWMT in later MDD. The CWMT improvement score also showed somewhat paradoxical associations to self-reported attention deficits, with gains being associated with poorer self-assessed cognition. Potential negative effects from CWMT underscore the importance of therapist-assisted interventions, psychoeducation, and/or broader interventions targeting functions beyond WM alone, in interventions targeting residual symptoms in remission from MDD. However, the small, strictly remitted sample, cautions firm conclusion regarding this.

### Strengths and Limitations

This is the first study to investigate how CWMT effects e-WM, PS, EF, and symptoms and different forms of rumination combined. Pre-registration of hypotheses was done before analysis. In addition, the sample was selected and the intervention comprehensive. Demand characteristics could have resulted in underreporting of symptoms and could thus have influenced expected associations between MADRS and PS. In addition, dropout was considerable, but not higher than comparable studies. To reduce the chance of type 2 errors, correction of multiple comparisons was not strictly implemented, increasing risk for type 1 errors. The small sample size makes correlation estimates unstable (Schönbrodt and Perugini, 2013), and novel findings in this study should be considered preliminary before being replicated in larger samples. However, it should be noted that this study has higher sample size than 75% of the studies in a recent meta-analysis of cognitive remediation in MDD (Legemaat et al., 2021).

This study shows some overlap with a pilot study on the acceptability and feasibility of CWMT (Hammar et al., 2020). Hammar et al. (2020) investigated some cognitive outcomes of CWMT, namely, WM, inhibition, and rumination, with the two latter similar to this study. However, analyses in the pilot study were done *before all the data were collected* on these measures. In addition, this study investigated several other relevant aspects of cognitive function: e-WM, EF, PS, and depressive symptoms, in a complete dataset. This study focused on the clinically relevant distinctions of hot cognition, PS, and EF, in addition to the differing perspective of investigating the mechanisms of interrelationships between cognitive function and symptoms pre- and post-CWMT. To the best of the authors knowledge, this study is the first CWMT study to do to so, supporting some previous findings (Joormann et al., 2011; Ronold et al., 2020a), while not others (Ronold et al., 2020b), regarding the association between cognitive functioning and depressive symptoms and rumination.

This study design, lacking control group and randomization, makes it hard to control for placebo effects/demand characteristics and retest effects in the sample. However, given the size of improvements, and associations between improvements in CWMT and cognition, it is suggested that both PS and EF are improved by this intervention. Large longitudinal double-blinded randomized controlled studies should replicate and improve on the findings, investigate to what degree effects are *clinically significant* beyond placebo effects, *transfer* to daily functioning, should investigate how long effects last, and what clinical implications they have for relapse, recurrence, quality of life and daily functioning in the remitted phase of MDD. In addition, the change scores utilized in the explorative hypotheses do not correct for the correlated nature of the pre- and post-measures and must be interpreted with caution (Hayes and Rockwood, 2017). Larger samples should thus investigate mediating effects of MDD history on the effects of CWMT, and the associations between EF PS and CWMT.

## Summary of Findings and Conclusions

The study supports that computerized working memory training improves cognition in remission from MDD. There was evidence for improvements in *some*, but not *all*, residual symptoms following MDD. Executive functions and processing speed improved, with largest effect for the former. Associations between emotional working memory for negative stimuli and rumination manifested initially but disappeared following the intervention. However, there was no significant decrease in rumination, nor depressive symptoms, but the sample showed low initial mood symptom load. Care should be taken in selection and personalization of interventions for optimal effects in studies and clinical settings. Interventions in remission should probably target a broad range of symptoms for optimal relapse prevention. Computerized working memory training appears to improve cognitive functions in remission from MDD and could thus potentially prevent new episodes and functional decline following MDD. Results suggest that computerized working memory training could be useful for improving processing speed, executive functions, and emotional working memory in MDD, but also that other interventions targeting symptoms could be implemented. Cognitive training targeting hot cognition could potentially remediate symptoms to a larger degree than CWMT. Future studies should investigate which individuals benefit from computerized working memory training and implement personalized interventions for residual symptoms and relapse prevention following MDD. Larger studies identifying subgroups that show different cognitive and symptomatic profiles could identify individuals who benefit from computerized working memory training, and should, together with larger, more controlled effect studies, be pursued by future research.

## Data Availability Statement

The raw data supporting the conclusions of this article will be made available by the authors, without undue reservation.

## Ethics Statement

The studies involving human participants were reviewed and approved by the Committee for Ethical Research in Western Norway (REK 2014/1079), in addition to the Norwegian Data Inspectorate. The participants provided their written informed consent to participate in this study.

## Author Contributions

ER wrote the current manuscript, collected data, and did statistical analyses. JJ designed the paradigm used to measure e-WM, contributed with ideas for design and analyses, and edited the manuscript. ÅH was PI of the study and contributed with, design, ideas and facilitated data collection, and edited the manuscript. All authors contributed to the article and approved the submitted version.

## Conflict of Interest

The authors declare that the research was conducted in the absence of any commercial or financial relationships that could be construed as a potential conflict of interest.

## Publisher's Note

All claims expressed in this article are solely those of the authors and do not necessarily represent those of their affiliated organizations, or those of the publisher, the editors and the reviewers. Any product that may be evaluated in this article, or claim that may be made by its manufacturer, is not guaranteed or endorsed by the publisher.

## References

[B1] Årdal G. Hammar Å. (2011). Is impairment in cognitive inhibition in the acute phase of major depression irreversible? Results from a 10-year follow-up study. Psychol. Psychother. 84, 141–150. 10.1348/147608310X502328 22903853

[B2] Ahern E. Bockting C. L. H. Semkovska M. (2019). A hot-cold cognitive model of depression: integrating the neuropsychological approach into the cognitive theory framework. Clin. Psychol. Eur. 1, 1–35. 10.32872/cpe.v1i3.34396

[B3] Ahern E. Semkovska M. (2017). Cognitive functioning in the first-episode of major depressive disorder: a systematic review and meta-analysis. Neuropsychology 31, 52–72. 10.1037/neu0000319 27732039

[B4] Aker M. Harmer C. Landrø I. I. (2014). More rumination and less effective emotion regulation in previously depressed women with preserved executive functions. BMC Psychiatry 14, 334. 10.1186/s12888-014-0334-4 25427967 PMC4253635

[B5] Allott K. Fisher C. A. Amminger G. P. Goodall J. Hetrick S. (2016). Characterizing neurocognitive impairment in young people with major depression: state, trait, or scar? Brain Behav. 6, 1–12. 10.1002/brb3.527 27781141 PMC5064339

[B6] Barkus E. (2020). Effects of working memory training on emotion regulation: transdiagnostic review. PsyCh. J. 9, 258–279. 10.1002/pchj.353 32166891

[B7] Bernhardt M. Klauke S. Schröder A. (2019). Longitudinal course of cognitive function across treatment in patients with MDD: a meta-analysis. J. Affect. Disord. 249, 52–62. 10.1016/j.jad.2019.02.021 30753954

[B8] Bourke C. Douglas K. Porter R. (2010). Processing of facial emotion expression in major depression: a review. Aust. N. Z. J. Psychiatry 44, 681–696. 10.3109/00048674.2010.496359 20636189

[B9] Cohen J. (1988). Statistical Power Analysis for the Behavioral Sciences, 2nd Edn. Hillsdale, NJ: Erlbaum.

[B10] Cunningham J. L. Wernroth L. Von Knorring L. Berglund L. Ekselius L. (2011). Agreement between physicians' and patients' ratings on the Montgomery-Åsberg Depression Rating Scale. J. Affect. Disord. 135, 148–153. [10.1016/j.jad.2011.07.005]10.1016/j.jad.2011.07.005 21856017 10.1016/j.jad.2011.07.005

[B11] Delis D. C. Kaplan E. Kramer J. H. (2001). Delis-Kaplan Executive Function System: Examiners Manual. San Antonio, TX: The Psychological Corporation.

[B12] Diamond A. (2013). Executive functions. Annu. Rev. Psychol. 64, 135-−168. 10.1146/annurev-psych-113011-143750 23020641 PMC4084861

[B13] Dotson V. M. McClintock S. M. Verhaeghen P. Kim J. U. Draheim A. A. Syzmkowicz S. M.. (2020). Depression and cognitive control across the lifespan: a systematic review and meta-analysis. Neuropsychol. Rev. 30, 461–476. 10.1007/s11065-020-09436-6 32385756 PMC9637269

[B14] Douglas K. M. Porter R. J. (2009). Longitudinal assessment of neuropsychological function in major depression. Aust. N Z. J. Psychiatry 43, 1105–1117. 10.3109/00048670903279887 20001409

[B15] Evans V. C. Iverson G. L. Yatham L. N. Lam R. W. (2014). The relationship between neurocognitive and psychosocial functioning in major depressive disorder: a systematic review. J. Clin. Psychiatry 75, 1359–1370. 10.4088/JCP.13r08939 25551235

[B16] Ferrari G. R. A. Vanderhasselt M. A. Rinck M. Demeyer I. Raedt R. De Beisel S.. (2021). A cognitive control training as add - on treatment to usual care for depressed inpatients. Cogn. Ther. Res. 45, 929–943. 10.1007/s10608-020-10197-y29908472

[B17] Figueroa C. A. DeJong H. Mocking R. J. T. Fox E. Rive M. M. Schene A. H.. (2019). Attentional control, rumination and recurrence of depression. J. Affect. Disorde. 256, 364–372. 10.1016/J.JAD.2019.05.072 31207560

[B18] Friedman N. P. Miyake A. (2017). Unity and diversity of executive functions: individual differences as a window on cognitive structure. Cortex, 86, 186–204. 10.1016/J.CORTEX.2016.04.023 27251123 PMC5104682

[B19] Gorlyn M. Keilp J. Burke A. Oquendo M. Mann J. J. Grunebaum M. (2015). Treatment-related improvement in neuropsychological functioning in suicidal depressed patients: paroxetine vs. bupropion. Psychiatry Res. 225, 407–412. 10.1016/j.psychres.2014.12.004 25555415 PMC4314330

[B20] Grinberg A. Egglefield D. A. Schiff S. Motter J. N. Sneed J. R. (2021). Computerized cognitive training: a review of mechanisms, methodological considerations, and application to research in depression. J. Cogn. Enhanc. 5, 359–371. 10.1007/s41465-021-00209-4

[B21] Groves S. J. Douglas K. M. Milanovic M. Bowie C. R. Porter R. J. (2021). Systematic review of the effects of evidence-based psychotherapies on neurocognitive functioning in mood disorders. Aust. N. Z. J. Psychiatry 55, 944–957. 10.1177/00048674211031479 34278831

[B22] Groves S. J. Douglas K. M. Porter R. J. (2018). A systematic review of cognitive predictors of treatment outcome in major depression. Front. Psychiatry 9, 382. 10.3389/fpsyt.2018.00382 30210368 PMC6121150

[B23] Gustavson D. E. Lurquin J. H. Michaelson L. E. Barker J. E. Carruth N. P. von Bastian C. C.. (2020). Lower general executive function is primarily associated with trait worry: a latent variable analysis of negative thought/affect measures. Emotion 20, 557–571. 10.1037/emo0000584 30816740 PMC6713624

[B24] Hammar Å. Ronold E. H. Årdal G. R. (2022). Cognitive impairment and neurocognitive profiles in major depression –a clinical perspective. Front. Psychiatry. 13, 764374. 10.3389/fpsyt.2022.764374 35345877 PMC8957205

[B25] Hammar Å. Semkovska M. Borgen I. M. H. Myklebost S. Ronold E. H. Sveen T.. (2020). A pilot study of cognitive remediation in remitted major depressive disorder patients. Appl. Neuropsychol. Adult 29, 172–182. 10.1080/23279095.2020.1726919 32088993

[B26] Hasselbalch B. J. Knorr U. Kessing L. V. (2011). Cognitive impairment in the remitted state of unipolar depressive disorder: a systematic review. J. Affect. Disorde. 134, 20–31. 10.1016/j.jad.2010.11.011 21163534

[B27] Hayes A. F. Rockwood N. J. (2017). Regression-based statistical mediation and moderation analysis in clinical research: observations, recommendations, and implementation. Behav. Res. Ther. 98, 39–57. 10.1016/j.brat.2016.11.00 27865431

[B28] Jandric S. Filakovic P. Kurtovic A. Kovac V. Benic D. Rogulja S.. (2021). The role of cognitive control and rumination in predicting depression among adolescents with internalizing disorders. Psychiatr. Danub. 33, 165–172. 10.24869/psyd.2021.165 34185737

[B29] Joormann J. Gotlib I. H. (2010). Emotion regulation in depression: relation to cognitive inhibition. Cogn. Emot. 24, 281–298. 10.1080/02699930903407948 20300538 PMC2839199

[B30] Joormann J. Levens S. M. Gotlib I. H. (2011). Sticky thoughts: depression and rumination are associated with difficulties manipulating emotional material in working memory. Psychol. Sci., 22, 979–983. 10.1177/0956797611415539 21742932 PMC11862919

[B31] Joormann J. Stanton C. H. (2016). Examining emotion regulation in depression: a review and future directions. Behav. Res. Ther. 86, 35–49. 10.1016/j.brat.2016.07.007 27492851

[B32] Keefe R. S. E. McClintock S. M. Roth R. M. Murali Doraiswamy P. Tiger S. Madhoo M. (2014). Cognitive effects of pharmacotherapy for major depressive disorder: a systematic review. J. Clin. Psychiatry 75, 864–876. 10.4088/JCP.13r08609 25099527

[B33] Kessing L. V. Andersen P. K. (2017). Evidence for clinical progression of unipolar and bipolar disorders. Acta Psychiatr. Scand. 135, 51–64. 10.1111/acps.12667 27858964

[B34] Koster E. H. W. Hoorelbeke K. (2015). Cognitive bias modification for depression. Curr. Opin. Psychol. 4, 119–123. 10.1016/j.copsyc.2014.11.012

[B35] Koster E. H. W. Hoorelbeke K. Onraedt T. Owens M. Derakshan N. (2017). Cognitive control interventions for depression: a systematic review of findings from training studies. Clin. Psychol. Rev. 53, 79–92. 10.1016/j.cpr.2017.02.002 28273486

[B36] Launder N. H. Minkov R. Davey C. G. Finke C. Gavelin H. M. Lampit A. (2021). Computerized cognitive training in people with depression: a systematic review and meta-analysis of randomized clinical trials. MedRxiv, 2021.03.23.21254003. Available online at http://medrxiv.org/content/early/2021/03/26/2021.03.23.21254003.abstract

[B37] Lee R. S. C. Hermens D. F. Porter M. A. Redoblado-Hodge M. A. (2012). A meta-analysis of cognitive deficits in first-episode Major Depressive Disorder. J. Affect. Disorde. 140, 113–124. 10.1016/j.jad.2011.10.023 22088608

[B38] Legemaat A. M. Semkovska M. Brouwer M. Geurtsen G. J. Burger H. Denys D.. (2021). Effectiveness of cognitive remediation in depression: a meta-analysis. Psychol. Med. 1–18. 10.1017/S0033291721001100. [Epub ahead of print]. 33849674 PMC9811271

[B39] Lengvenyte A. Coppola F. Jaussent I. Courtet P. Olié E. (2020). Improved functioning following computerized working memory training (COGMED®) in euthymic patients with bipolar disorder and cognitive complaints: an exploratory study. J. Affect. Disorde. 262, 414–421. 10.1016/j.jad.2019.11.062 31740107

[B40] Liu H. Funkhouser C. J. Langenecker S. A. Shankman S. A. (2021). Set shifting and inhibition deficits as potential endophenotypes for depression. Psychiatry Res. 300, 113931. 10.1016/j.psychres.2021.113931 33894683 PMC8141023

[B41] Lundqvist D. Flykt A. Öhman A. (1998). The Karolinska Directed Emotional Faces - KDEF, CD ROM from Department of Clinical Neuroscience, Psychology section, Karolinska Institutet, ISBN 91-630-7164-9

[B42] Majer M. Ising M. Künzel H. Binder E. B. Holsboer F. Modell S.. (2004). Impaired divided attention predicts delayed response and risk to relapse in subjects with depressive disorders. Psychol. Med. 34, 1453–1463. 10.1017/S0033291704002697 15724876

[B43] McCabe D. P. Roediger H. L. McDaniel M. A. Balota D. A. Hambrick D. Z. (2010). The relationship between working memory capacity and executive functioning: evidence for a common executive attention construct. Neuropsychology 24, 222–243. 10.1037/a0017619 20230116 PMC2852635

[B44] McDermott L. M. Ebmeier K. P. (2009). A meta-analysis of depression severity and cognitive function. J. Affect. Disorde. 119, 1–8. 10.1016/j.jad.2009.04.022 19428120

[B45] McIntyre R. S. Cha D. S. Soczynska J. K. Woldeyohannes H. O. Gallaugher L. A. Kudlow P.. (2013). Cognitive deficits and functional outcomes in major depressive disorder: determinants, substrates, and treatment interventions. Depress. Anxiety, 30, 515–527. 10.1002/da.22063 23468126

[B46] McLennan S. N. Mathias J. L. (2010). The depression-executive dysfunction (DED) syndrome and response to antidepressants: a meta-analytic review. Int. J. Geriatr. Psychiatry 25, 933–944. 10.1002/gps.2431 20872927

[B47] Miskowiak K. W. Seeberg I. Jensen M. B. Balanzá-Martínez V. del Mar Bonnin C. Bowie C. R.. (2022). Randomised controlled cognition trials in remitted patients with mood disorders published between 2015 and 2021: A systematic review by the international society for bipolar disorders targeting cognition task force. Bipolar Disord. 1–21. 10.1111/bdi.13193 35174594 PMC9541874

[B48] Miyake A. Friedman N. P. Emerson M. J. Witzki A. H. Howerter A. Wager T. D. (2000). The unity and diversity of executive functions and their contributions to complex “Frontal Lobe” tasks: a latent variable analysis. Cogn. Psychol. 41, 49–100. 10.1006/cogp.1999.0734 10945922

[B49] Montgomery S. A. Åsberg M. (1979). A new depression scale designed to be sensitive to change. Br. J. Psychiatry 134, 382–389. 10.1192/bjp.134.4.382 444788

[B50] Motter J. N. Grinberg A. Lieberman D. H. Iqnaibi W. B. Sneed J. R. (2019). Computerized cognitive training in young adults with depressive symptoms: effects on mood, cognition, and everyday functioning. J. Affect. Disorde. 245, 28–37. 10.1016/j.jad.2018.10.109 30366235

[B51] Motter J. N. Pimontel M. A. Rindskopf D. Devanand D. P. Doraiswamy P. M. Sneed J. R. (2016). Computerized cognitive training and functional recovery in major depressive disorder: a meta-analysis. J. Affect. Disorde. 189, 184–191. 10.1016/j.jad.2015.09.022 26437233

[B52] Mueller T. I. Leon A. C. Keller M. B. Solomon D. A. Endicott J. Coryell W.. (2013). Recurrence after recovery from major depressive disorder during 15 years of observational follow-up. Depression Sci. Mental Health 6, 100–106. 10.1176/ajp.156.7.1000 10401442

[B53] Myklebost S. B. Nordgreen T. Hammar Å. (2021). Applied neuropsychology : adult an open pilot study of an internet-delivered intervention targeting self-perceived residual cognitive symptoms after major depressive disorder. Appl. Neuropsychol. Adult 1–10. 10.1080/23279095.2021.1901706. [Epub ahead of print]. 33813984

[B54] Nafilyan V. Pabon M. A. de Coulon A. (2021). The Causal Impact of Depression on Cognitive Functioning: evidence from Europe. SSRN, 14049, 1–40. 10.2139/ssrn.377173224923362

[B55] Nakagawa S. (2004). A farewell to Bonferroni: the problems of low statistical power and publication bias. Behav. Ecol. 15, 1044–1045. 10.1093/beheco/arh107

[B56] Nigg J. T. Jester J. M. Stavro G. M. Ip K. I. Puttler L. I. Zucker R. A. (2017). Specificity of executive functioning and processing speed problems in common psychopathology. Neuropsychology 31, 448–466. 10.1037/neu0000343 28094999 PMC5408314

[B57] Nolen-Hoeksema S. Wisco B. E. Lyubomirsky S. (2008). Rethinking rumination. Perspect. Psychol. Sci. 3, 400–424. 10.1111/j.1745-6924.2008.00088.x 26158958

[B58] Nuño L. Gómez-Benito J. Carmona V. R. Pino O. (2021). A systematic review of executive function and information processing speed in major depression disorder. Brain Sci. 11, 1–18. 10.3390/brainsci11020147 33499360 PMC7912411

[B59] Paelecke-Habermann Y. Pohl J. Leplow B. (2005). Attention and executive functions in remitted major depression patients. J. Affect. Disorde. 89(1–3), 125–135. 10.1016/j.jad.2005.09.006 16324752

[B60] Parkinson W. L. Rehman Y. Rathbone M. Upadhye S. (2020). Performances on individual neurocognitive tests by people experiencing a current major depression episode: a systematic review and meta-analysis. J. Affect. Disorde. 276, 249–259. 10.1016/j.jad.2020.07.036 32697706

[B61] Pearson (2022). Research Evidence for the Improvement of Working Memory and Attention. Available online at: https://download.cogmed.com/claims_and_evidence (accessed February 2, 2022).

[B62] Porter R. J. Robinson L. J. Malhi G. S. Gallagher P. (2015). The neurocognitive profile of mood disorders - a review of the evidence and methodological issues. Bipolar Disorders 17, 21–40. 10.1111/bdi.12342 26688288

[B63] Pu S. Noda T. Setoyama S. Nakagome K. (2018). Empirical evidence for discrete neurocognitive subgroups in patients with non-psychotic major depressive disorder: clinical implications. Psychol. Med. 48, 2717–2729. 10.1017/S003329171800034X 29679991

[B64] Rock P. L. Roiser J. P. Riedel W. J. Blackwell A. D. (2014). Cognitive impairment in depression: a systematic review and meta-analysis. Psychol. Med. 44, 2029–2040. 10.1017/S0033291713002535 24168753

[B65] Roiser J. P. Sahakian B. J. (2013). Hot and cold cognition in depression. CNS Spectr. 18, 139–149. 10.1017/S1092852913000072 23481353

[B66] Ronold E. Schmid M. T. Hammar Å. (2021). Risk factors and cognitive deficits in first episode major depression: a five-year longitudinal study of explorative subgroups. Biol. Psychiatry 89, S131. 10.1016/j.biopsych.2021.02.338

[B67] Ronold E. H. Joormann J. Hammar Å. (2020a). Facing recovery: emotional bias in working memory, rumination, relapse, and recurrence of major depression; an experimental paradigm conducted five years after first episode of major depression. Appl. Neuropsychol. Adult 27, 299–310. 10.1080/23279095.2018.1550406 30646773

[B68] Ronold E. H. Schmid M. T. Oedegaard K. J. Hammar Å. (2020b). A longitudinal 5-year follow-up study of cognitive function after first episode major depressive disorder: exploring state, scar and trait effects. Front. Psychiatry 11, 1395. 10.3389/fpsyt.2020.575867 33364989 PMC7750430

[B69] Rosenblat J. D. Kakar R. McIntyre R. S. (2015). The cognitive effects of antidepressants in major depressive disorder: a systematic review and meta-analysis of randomized clinical trials. Int. J. Neuropsychopharmacol. 19, 1–13. 10.1093/ijnp/pyv082 26209859 PMC4772818

[B70] Russo M. Mahon K. Burdick K. E. (2015). Measuring cognitive function in MDD: emerging assessment tools. Depress. Anxiety 32, 262-−269. 10.1002/da.22297 25421437 PMC4407945

[B71] Salthouse T. A. (1996). The processing-speed theory of adult age differences in cognition. Psychol. Rev. 103, 403–428. 10.1037/0033-295X.103.3.403 8759042

[B72] Scharfen J. Blum D. Holling H. (2018). Response time reduction due to retesting in mental speed tests: a meta-analysis. J. Intell. 6, 1–28. 10.3390/jintelligence6010006 31162433 PMC6480749

[B73] Schmid M. Hammar Å. (2021). First-episode patients report cognitive difficulties in executive functioning 1 year after initial episode of major depressive disorder. Front. Psychiatry 12, 1–10. 10.3389/fpsyt.2021.667238 34135786 PMC8200526

[B74] Schmid M. T. Hammar Å. (2013). A follow-up study of first episode major depressive disorder. Impairment in inhibition and semantic fluency-potential predictors for relapse? Front. Psychol. 4, 633. 10.3389/fpsyg.2013.00633 24062714 PMC3772336

[B75] Schönbrodt F. D. Perugini M. (2013). At what sample size do correlations stabilize? J. Res. Personality 47, 609–612. 10.1016/j.jrp.2013.05.009

[B76] Schwert C. Aschenbrenner S. Weisbrod M. Schröder A. (2017). Cognitive impairments in unipolar depression: the impact of rumination. Psychopathology 50, 347–354. 10.1159/000478785 28850956

[B77] Semkovska M. Quinlivan L. O'Grady T. Johnson R. Collins A. O'Connor J.. (2019). Cognitive function following a major depressive episode: a systematic review and meta-analysis. Lancet Psychiatry 6, 851–861. 10.1016/S2215-0366(19)30291-3 31422920

[B78] Sheehan D. V. Lecrubier Y. Sheehan K. H. Amorim P. Janavs J. Weiller E.. (1998). The Mini-International Neuropsychiatric Interview (M.I.N.I.): the development and validation of a structured diagnostic psychiatric interview for DSM-IV and ICD-10. J. Clin. Psychiatry 59, 22–33. 9881538

[B79] Snyder H. R. (2013). Major depressive disorder is associated with broad impairments on neuropsychological measures of executive function: a meta-analysis and review. Psychol. Bull. 139, 81–132. 10.1037/a0028727 22642228 PMC3436964

[B80] Snyder H. R. Friedman N. P. Hankin B. L. (2020). Associations between task performance and self-report measures of cognitive control : shared versus distinct abilities. Assessment 28, 1080–1096. 10.1177/1073191120965694 33084353 PMC8058111

[B81] Snyder H. R. Miyake A. Hankin B. L. (2015). Advancing understanding of executive function impairments and psychopathology: bridging the gap between clinical and cognitive approaches. Front. Psychol. 6, 328. 10.3389/fpsyg.2015.00328 25859234 PMC4374537

[B82] Sociali A. Borgi M. Pettorruso M. Di Carlo F. Di Natale C. Tambelli A.. (2022). What role for cognitive remediation in the treatment of depressive symptoms? A superiority and noninferiority meta-analysis for clinicians. Depress. Anxiety. 1–21. 10.1002/da.23263. [Epub ahead of print]. 35536033

[B83] Solomon D. A. Keller M. B. Leon A. C. Mueller T. I. Lavori P. W. Shea M. T.. (2000). Multiple recurrences of major depressive disorder. Am. J. Psychiatry 157, 229–233. 10.1176/appi.ajp.157.2.229 10671391

[B84] Stroop J. R. (1935). Studies of interference in serial verbal reactions. J Exp. Psychol. 18, 643–662. 10.1037/h0054651

[B85] Sumiyoshi T. Watanabe K. Noto S. Sakamoto S. Moriguchi Y. Hammer-Helmich L.. (2021). Relationship of subjective cognitive impairment with psychosocial function and relapse of depressive symptoms in patients with major depressive disorder: analysis of longitudinal data from perform-j. Neuropsychiatric Dis. Treat. 17, 945–955. 10.2147/NDT.S288108 33814911 PMC8009536

[B86] Svanborg P. Åsberg M. (2001). A comparison between the Beck Depression Inventory (BDI) and the self-rating version of the Montgomery Asberg Depression Rating Scale (MADRS). J. Affect. Disord. 64, 203–216. 10.1016/s0165-0327(00)00242-1 11313087

[B87] Taylor M. M. Snyder H. R. (2021). Repetitive negative thinking shared across rumination and worry predicts symptoms of depression and anxiety. J. Psychopathol. Behav. Assess. 43, 904–915. 10.1007/s10862-021-09898-9

[B88] Timm C. Ubl B. Zamoscik V. Ebner-Priemer U. Reinhard I. Huffziger S.. (2017). Cognitive and affective trait and state factors influencing the long-Term symptom course in remitted depressed patients. PLoS One, 12, 1–16. 10.1371/journal.pone.0178759 28575049 PMC5456349

[B89] Trapnell P. D. Campbell J. D. (1999). Private self-consciousness and the five-factor model of personality: distinguishing rumination from reflection. J. Pers. Soc. Psychol. 76, 284–304. 10.1037/0022-3514.76.2.284 10074710

[B90] Treynor W. Gonzalez R. Nolen-Hoeksema S. (2003). Rumination reconsidered: a psychometric analysis. Cognit. Ther. Res. 27, 247–259. 10.1023/A:1023910315561

[B91] Vălenaş S. P. Szentágotai-Tătar A. (2017). The relationship between rumination and executive functions: a meta-analysis. J. Evid. Based Psychother. 17, 23–52. 10.24193/jebp.2017.2.227378739

[B92] Watkins E. R. Roberts H. (2020). Reflecting on rumination: consequences, causes, mechanisms and treatment of rumination. Behav. Res. Ther. 127, 103573. 10.1016/j.brat.2020.103573 32087393

[B93] Webb S. L. Loh V. Lampit A. Bateman J. E. Birney D. P. (2018). Meta-analysis of the effects of computerized cognitive training on executive functions: a cross-disciplinary taxonomy for classifying outcome cognitive factors. Neuropsychol. Rev. 28, 232–250. 10.1007/s11065-018-9374-8 29721646

[B94] Wechsler D. (1999). Wechsler Abbreviated Scale of Intelligence (WASI). San Antonio, TX: Psychological Corporation.

[B95] Woolf C. Lampit A. Norrie Z. S. J. S. L. M. Burke D. Naismith S. L. (2021). A systematic review and meta - analysis of cognitive training in adults with major depressive disorder. Neuropsychol. Rev. 32, 419–437. 10.1007/s11065-021-09487-3 33913064

[B96] World Medical Association (2013). World Medical Association Declaration of Helsinki: ethical principles for medical research involving human subjects. JAMA. 310, 2191–2194. 10.1001/jama.2013.281053 24141714

[B97] Yang Y. Cao S. Shields G. S. Teng Z. Liu Y. (2017). The relationships between rumination and core executive functions: a meta-analysis. Depress. Anxiety 34, 37–50. 10.1002/da.22539 27378739

[B98] Zajecka J. M. (2013). Residual symptoms and relapse: mood, cognitive symptoms, and sleep disturbances. J. Clin. Psychiatry 74(SUPPL. 2), 9–13. 10.4088/JCP.12084su1c.02 24191972

[B99] Zetsche U. Bürkner P. C. Schulze L. (2018). Shedding light on the association between repetitive negative thinking and deficits in cognitive control – a meta-analysis. Clin. Psychol. Rev. 63, 56–65. 10.1016/j.cpr.2018.06.001 29913351

